# Complete Androgen Insensitivity Syndrome in Three Sisters

**Published:** 2013-12-22

**Authors:** Levent Verim

**Affiliations:** Department of Urology, Haydarpasa Numune Training Hospital, Istanbul, Turkey

**Keywords:** Disorder of Sexual Development, 46 XY Female, Androgen Receptor, Mutation, Infertility

## Abstract

Disorders of sexual development (DSD) are congenital anomalies due to atypical development of chromosomes, gonads and anatomy. Complete androgen insensitivity syndrome (CAIS), also known as testicular feminization (TF) is a rare DSD disease. The majority of CAIS patients apply to hospital with the complaint of primary amenorrhea or infertility. Given that CAIS patients are all phenotypically female while having 46, XY karyotypes, CAIS diagnosis should be disclosed in an age-appropriate manner preferably by a mental health professional. Cases are reported here for three 46XY siblings consistent with CAIS.

## Introduction

Androgen Insensitivity syndrome (AIS) is a disorder of sexual development (DSD) formerly classified as male pseudo hermaphroditism and referred to as XY DSD. AIS is caused by a mutation in the androgen receptor (AR) gene resulting in deficient action of androgens and therefore incomplete masculinization. Two forms of AIS are described: complete androgen insensitivity syndrome (CAIS) and partial androgen insensitivity syndrome (PAIS). CAIS is also known as testicular feminization (TF). Patients with CAIS are all phenotypically female while having 46, XY karyotype and testis, and sometimes first come to medical attention with complaints of amenorrhea or infertility. Explaining the nature of their DSD, including infertility, to these women is a delicate matter for medical professionals. Cases are reported here for three 46XY siblings consistent with CAIS (1).

## Case Report

### Case 1

A 19-year-old girl was admitted to gynecology clinic with the complaint of primary amenorrhea. The patient appeared phenotypically female and was raised by her parents as a girl. She refused colposcopy because of being virgin. On physical examination, her external genitalia and breast development appeared as completely normal feminine structures but pubic and axillary hair was absent. There were bilateral palpable masses in the inguinal regions.

**Fig 1:**
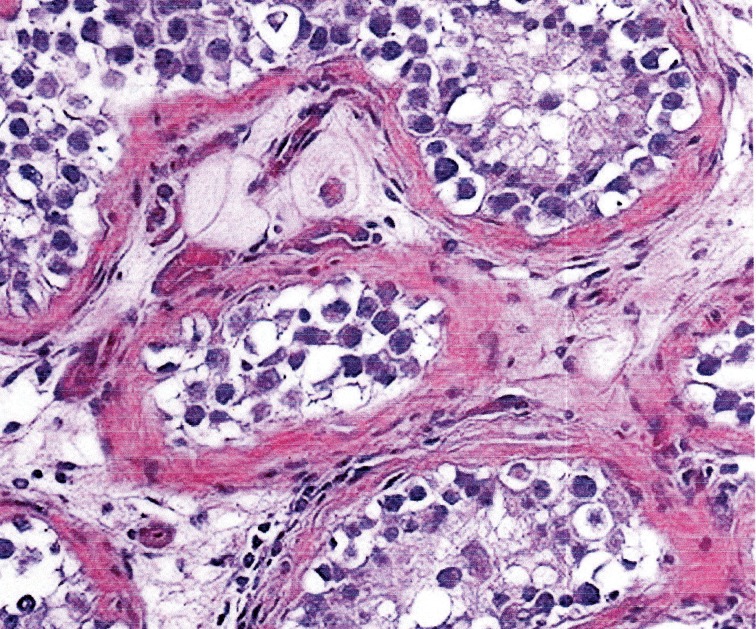
Sertoli cell adenomas and seminiferous tubles with intratubular germ cell neoplasia showing large cells with enlarged vesicular nuclei in the clear cytoplasm.

Trans abdominal ultrasonography revealed inguinal masses consistent with immature testes. These gonads were 36×15 mm and 33×10 mm in size at right and left side respectively. Uterus and ovaries were not detected. The patient was referred to the endocrinology department for further investigation. Her routine blood chemistries were within normal limits, luteinizing hormone (LH) level was slightly elevated and cytogenetic analysis showed a 46, XY karyotype. Her bone age was compatible with her actual age. In light of all the findings, the most probable diagnosis was considered to be CAIS. The patient was consulted with a psychologist about her DSD and was then referred to urology clinic for bilateral gonadectomy because she was post-pubertal and adequate feminization in response to aromatization of testicular androgen. Orchiectomy was accomplished on both sides and the patient was discharged without complication on third postoperative day. Multiple Sertoli cell adenomas and intratubulary germ cell neoplasia were revealed based on the histopathology of testes. The presence of Leydig cell hyperplasia and fibrosis made the diagnosis compatible with CAIS (Fig 1). Abdominal and thorax CT imagings taken after surgery showed neither abnormality nor metastatic disease. Long-term hormonal (estradiol) replacement therapy was prescribed by the endocrinology department because of a low level of plasma estradiol after castration.

### Case 2

**Fig 2:**
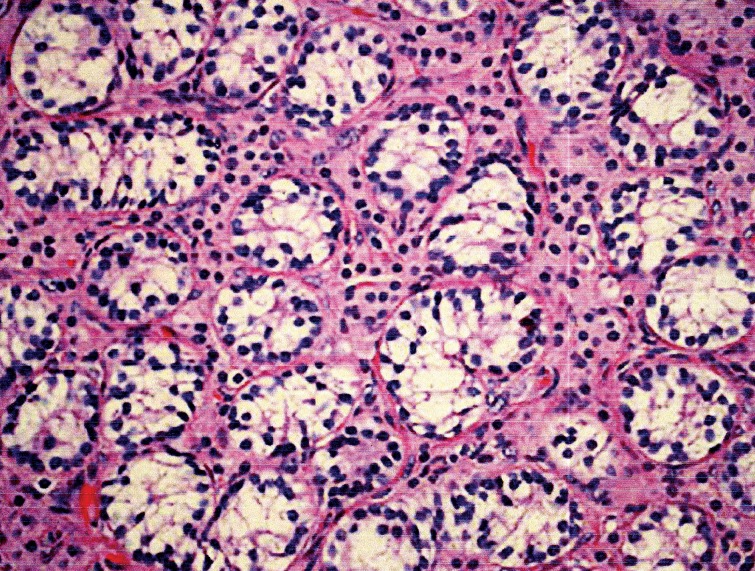
Histopathology of testicular tissue showing immature germ cells within atrophic seminiferous tubules and hyperplasia of leydig cells.

A 22-year-old woman referred to endocrinology and gynecology clinics soon after the operation on her younger sister (Case 1). Her medical history was similar to that of her sister with the symptom of primary amenorrhea. She was recently married and described no sexual problem during intercourse. She had full breast development and feminine appearance of external genitalia with sparse pubic hair. A long and blind ending vagina was found in colposcopy. There were bilateral inguinal mobile masses on palpation that resembled testes on ultrasonography. Neither uterus nor were ovaries demonstrated on the scanning of the abdomen with ultrasonography. Her karyotype was 46, XY and the level of testosterone in peripheral blood was higher than the normal female range. The other biochemical measurements were within normal limits. The patient was diagnosed as CAIS like her 19-year-old sister and her disease was explained to her with the help of a psychologist. Bilateral inguinal orchiectomy was performed in urology clinic and she was discharged at second postoperative day without complication. Histopathologic report of surgical specimen was Sertoli cell adenomas with atrophic seminiferous tubules and Leydig cell hyperplasia. Some of the tubules had thickened basement membranes filled with hyaline substance with very rare spermatogonium (Fig 2). There were no sign of epididymis or efferent ductules in the whole specimen. Estradiol replacement therapy was also prescribed after gonadectomy.

### Case 3

A 27-year-old woman, older sister of cases 1 and 2, and married for a long time with no children. She was also invited to our hospital via phone call. She later brought her discharge report given from another hospital about her DSD. She had been operated 6 years previously because of bilateral inguinal masses. Hernia repairment and bilateral orchiectomy had been performed in the same session. According to the histopathology report, germ cell aplasia, focal tubulary atrophia, hyalinization of peritubulary area and widespread Leydig cell hyperplasia were described in the histopathology of specimen. She had a 46, XY karyotype like her sisters. She had been informed about CAIS and about being barren, however, her relatives were not informed about her diagnosis. She refused physical re-examination in our clinic.

## Discussion

DSD’s are congenital anomalies due to atypical development of chromosomes, gonads and anatomy. A new nomenclature of DSD was proposed instead of the terms such as 'pseudohermaphroditism', 'hermaphroditism', 'intersex', 'sex reversal', or 'ambiguous gender' which are often considered pejorative by patients (1). The incidence of DSD is estimated as one in 5, 500 live births. Congenital adrenal hyperplasia (CAH) is the most common cause of DSD. The incidence of CAH is one in 15 000 (2). AIS is the variant of DSD defined by Morris (3). CAIS is a rare form of AIS and the prevalence is estimated at between one in 20000 and one in 60000 live births. CAIS is rarely recognized at infancy because of the female phenotype. Our patients had complete female phenotype but we were unable to obtain photographs of their bodily features because of their views of religion. It should also be noted that even when religious views do not preclude obtaining such photographs, some individuals with DSD subsequently describe the photography experience as humiliating (4). The majority of CAIS patients admitted to hospital with the complaint of primary amenorrhea and occasionally diagnosed at the time of surgery for an inguinal hernia containing a testis. Newborn girls with an inguinal hernia, most probably have CAIS and should undergo a prompt karyotype analysis. CAIS is inherited in an X-linked recessive manner and characterized by resistance to androgen due to a mutation in the *AR* gene. *AR* gene is a proteincoding gene located at Xq11.2-q12, and codes for a protein that functions as a steroid hormone-activated transcription factor. Mutation in the *AR* gene causes androgen unresponsiveness which then affects transactivation of androgen-responsive genes in peripheral target cells. Androgen is a group of sex-steroid hormones responsible for male sex differentiation, development of male external genitalia and subsequent masculinizing puberty.

Androgens are produced by testes, ovaries (in lesser amounts) and the adrenal glands in both sexes. Testosterone, an androgen, is converted to the more potent androgen, dihydrotestosterone by the action of 5 α-reductase in target tissues. The mutation of *AR* gene causes the failing of this cascade and ends with incomplete masculinization and/or CAIS. More than 400 different mutations of AR gene causing CAIS have been reported. But only 23-78% of the PAIS cases had an *AR* gene mutation. PAIS may reveal as apparently normal male except for infertility (infertile male syndrome) or characterized by external ambiguous genitalia with normal development of epididymis, vas deferens and seminal vesicles (Reifenstein syndrome, 5-8).

In the differential diagnosis; 17 β-hydroxysteroid dehydrogenase type3 deficiency (17 β HD-3) is the most common XY DSD mimicking CAIS. The external genitalia is phenotypically female. 17 β HD-3 deficiency is an autosomal recessive transmitted disease with 46XY karyotype. The transformation of androstenedione into testosterone is impaired in 17 β HD-3.

The presence of normal epididymis, vas deferens, seminal vesicles, male voice, pubic and axillary hair growth and sometimes clitoromegaly are important diagnostic clues for differentiating 17 β HD-3 deficiency from CAIS. The prostate is absent because of ineffective level of androgen. Another DSD disease which mimics CAIS is Swyer syndrome also called XY gonadal dysgenesis and caused by mutation of the sex-determining (SRY) gene on the Y chromosome. Swyer syndrome can be differentiated from CAIS with the lack of breast development, presence of uterus in the imaging studies and presence of complete pubic and axillary hair. Mayer-Rokitansky-Küster- Hauser (MRKH) syndrome or Müllerian agenesis is a disease which can mimic CAIS lack of primary amenorrhea, underdeveloped vagina and normal breast development. However, the 46, XX karyotype is critical in making the diagnostic distinction. Testosterone level in MRKH syndrome is in normal range but elevated in CAIS (9, 10). Elevated testosterone levels in CAIS patient serve as a substrate for estogen synthesis which results in further feminization at prepubertal period. Furthermore dysgenetic male gonads in CAIS tend to undergo malignant transformation and possibility of malignancy increases remarkably with age. If diagnosis of CAIS is established, surgical castration should be applied by age 20 because of the risk of testicular cancer.

The risk is 1% when the testis is retained in the inguinal canal after puberty and half of the testicular neoplasms are malignant (dysgerminoma), but incidence of germ cell malignancy is shown to be as low as 0.8% before puberty. In patients who develop virilization and have a XY karyotype, the gonads should be removed immediately to preserve the female phenotype and female gender identity. The patients with CAIS should be followed up after gonadectomy as they have the signs and symptoms of postmenopausal woman. Therefore oral conjugated estrogen or transdermal estrogen should be administered for relieving these symptoms (11). Long-term studies indicate that with appropriate medical and psychological treatment, women with CAIS can be satisfied with their sexual function and psychosexual development. However, it is difficult for many healthcare professionals who lack expertise in this particular area to explain to a woman that she has a male Y chromosome and is infertile especially in certain cultures (12). This explanation should be carefully nuanced. For example, if a woman is told that she is "genetically male" she may understand that she is in reality a male and therefore a man. The ethical principle guiding the medical practitioners is to treat each patient with maximum benefit for that person. Informing the patient about her genetic constitution still remains a debate among some healthcare providers. Although Money (13) recommended full disclosure by the time a child completed high school unless there were significant cognitive limitations, our experience is that other clinicians frequently advised permanent withholding of disclosure from the patient, and sometimes even from the parent. The argument is that disclosure would lead to unacceptable harm to a fragile patient. If a policy of non-disclosure is maintained, she might never discover the truth about her genetic identity and her infertility. Unquestionably, the greatest harm would result if the patient found out about her diagnosis by modern information technology in an unmonitored setting where psychological assistance is not immediately available. The harm associated with disclosure can be minimized by a sensitive and skillful approach to the adolescent and adult patient. If the patient is a child, diagnosis should be disclosed in an age-appropriate manner and in conjunction psychoeducation about the underlying biological condition, preferably by a mental health professional along with a psychologist (14-18).
